# Enzyme Replacement Therapy with Pabinafusp Alfa for Neuronopathic Mucopolysaccharidosis II: An Integrated Analysis of Preclinical and Clinical Data

**DOI:** 10.3390/ijms222010938

**Published:** 2021-10-10

**Authors:** Roberto Giugliani, Ana Maria Martins, Torayuki Okuyama, Yoshikatsu Eto, Norio Sakai, Kimitoshi Nakamura, Hideto Morimoto, Kohtaro Minami, Tatsuyoshi Yamamoto, Mariko Yamaoka, Toshiaki Ikeda, Sairei So, Kazunori Tanizawa, Hiroyuki Sonoda, Mathias Schmidt, Yuji Sato

**Affiliations:** 1 Department of Genetics, Hospital de Clínicas de Porto Alegre, Universidade Federal do Rio Grande do Sul, Porto Alegre 90040-060, Brazil; rgiugliani@hcpa.edu.br; 2 Reference Center in Inborn Errors of Metabolism, Universidade Federal de São Paulo, São Paulo 04021-001, Brazil; ni.martins10@gmail.com; 3 Center for Lysosomal Storage Diseases, National Centre for Child Health and Development, Tokyo 157-8535, Japan; okuyama-t@ncchd.go.jp; 4 Advanced Clinical Research Centre & Asian Lysosome Storage Disorder Centre, Institute of Neurological Disorders, Kanagawa 215-0026, Japan; yosh@sepia.ocn.ne.jp; 5 Division of Health Sciences, Osaka University Graduate School of Medicine, Osaka 565-0871, Japan; norio@sahs.med.osaka-u.ac.jp; 6 Department of Pediatrics, Kumamoto University Graduate School of Medical Science, Kumamoto 860-8556, Japan; nakamura@kumamoto-u.ac.jp; 7 JCR Pharmaceuticals, Hyogo 659-0021, Japan; morimoto-h@jcrpharm.co.jp (H.M.); minami-k@jcrpharm.co.jp (K.M.); t-yamamoto@jcrpharm.co.jp (T.Y.); m-yamaoka@jcrpharm.co.jp (M.Y.); ikeda-t@jcrpharm.co.jp (T.I.); sou-s@jcrpharm.co.jp (S.S.); tanizawa-k@jcrpharm.co.jp (K.T.); sonoda-h@jcrpharm.co.jp (H.S.); mschmidt@jcrpharm.co.jp (M.S.)

**Keywords:** neuronopathic mucopolysaccharidosis, Hunter syndrome, mucopolysaccharidosis II, iduronate-2-sulfatase, enzyme replacement therapy, neurodegeneration, neurocognitive impairment, pabinafusp alfa, blood–brain barrier

## Abstract

Enzyme replacement therapy (ERT) improves somatic manifestations in mucopolysaccharidoses (MPS). However, because intravenously administered enzymes cannot cross the blood–brain barrier (BBB), ERT is ineffective against the progressive neurodegeneration and resultant severe central nervous system (CNS) symptoms observed in patients with neuronopathic MPS. Attempts to surmount this problem have been made with intrathecal and intracerebroventricular ERT in order to achieve CNS effects, but the burdens on patients are inimical to long-term administrations. However, since pabinafusp alfa, a human iduronate-2-sulfatase fused with a BBB-crossing anti-transferrin receptor antibody, showed both central and peripheral efficacy in a mouse model, subsequent clinical trials in a total of 62 patients with MPS-II (Hunter syndrome) in Japan and Brazil substantiated this dual efficacy and provided an acceptable safety profile. To date, pabinafusp alfa is the only approved intravenous ERT that is effective against both the somatic and CNS symptoms of patients with MPS-II. This article summarizes the previously obtained preclinical and clinical evidence related to the use of this drug, presents latest data, and discusses the preclinical, translational, and clinical challenges of evaluating, ameliorating, and preventing neurodegeneration in patients with MPS-II.

## 1. Introduction

A number of treatment modalities have been developed for patients with mucopolysaccharidoses (MPS). Chief among them is enzyme replacement therapy (ERT), which compensates the specific genetic deficiencies of enzymes and thereby ameliorates most of the somatic symptoms caused by the systemic accumulation of glycosaminoglycans (GAGs) and related events [1,2]. However, as large molecules cannot penetrate the blood–brain barrier (BBB), intravenously (IV) administered enzymes are prevented from reaching the brain parenchyma and catabolizing the GAGs therein. The GAG accumulation that then forms in the central nervous system (CNS) initiates a complex neurodegenerative process [3] that culminates in multifaceted progressive CNS symptoms in patients with MPS I, II, III, and VII (also known as neuronopathic MPS), often leading to early mortality [4].

Various efforts have been made to address the debilitating CNS manifestations seen in patients with neuronopathic MPS, including hematopoietic stem cell transplantation, gene therapy, and ERT via intrathecal (IT) and intracerebroventricular (ICV) administration. IT and ICV ERT are intended to circumvent the BBB and deliver enzymes directly into the brain, and they have been reported to show a positive CNS efficacy [5,6,7]. However, they invariably involve invasive procedures that are not conducive to long-term repeated administrations, and because they are ineffective against the somatic symptoms patients are faced with the additional onus of concomitant IV ERT.

Other attempts to address CNS symptoms have included utilizing the insulin [8,9] and transferrin [10] receptors located on the cerebrovascular endothelial cells so that the modified enzymes can traverse the BBB through these receptors and exert effects on the brain parenchyma.

Pabinafusp alfa (JR-141), developed by JCR Pharmaceuticals, consists of human iduronate-2-sulfatase (IDS), the enzyme in which patients with MPS-II are deficient (Hunter syndrome), fused to the C-terminus of the heavy chain of an anti-human transferrin receptor (hTfR) antibody. Its successful delivery across the BBB into the CNS by way of TfR-mediated transcytosis has been demonstrated in animal models, along with the resultant effects of decreasing heparan sulfate (HS) accumulations in the brain [11,12]. These promising preclinical data prompted the first human phase I/II study in Japan involving patients with MPS-II, which also produced encouraging results [13]. The subsequent phase II study in Brazil [14] and a phase II/III study in Japan [15] further substantiated the somatic/peripheral and central efficacy of pabinafusp alfa, leading to its regulatory approval for general use in Japan in March 2021 as the first BBB-crossing ERT. This article reports an integrated analysis of the hitherto accumulated preclinical and clinical data, including the latest long-term efficacy and safety data from the ongoing extension studies in the two countries, as well as some of the methodological and scientific challenges that had to be overcome in preclinical and clinical evaluations of the drug’s efficacy against complex progressive neurodegeneration.

## 2. Results

### 2.1. Preclinical Safety and Efficacy Results

#### 2.1.1. Mechanism of Action: Cellular Uptake and BBB Penetration

Pabinafusp alfa has mannose-6-phosphate (M6P) residues that possess binding affinity against M6P receptors and is taken up by target cells through M6P receptor-mediated endocytosis; the TfR-mediated pathway also facilitates cellular uptake [11].

**Figure 1:**
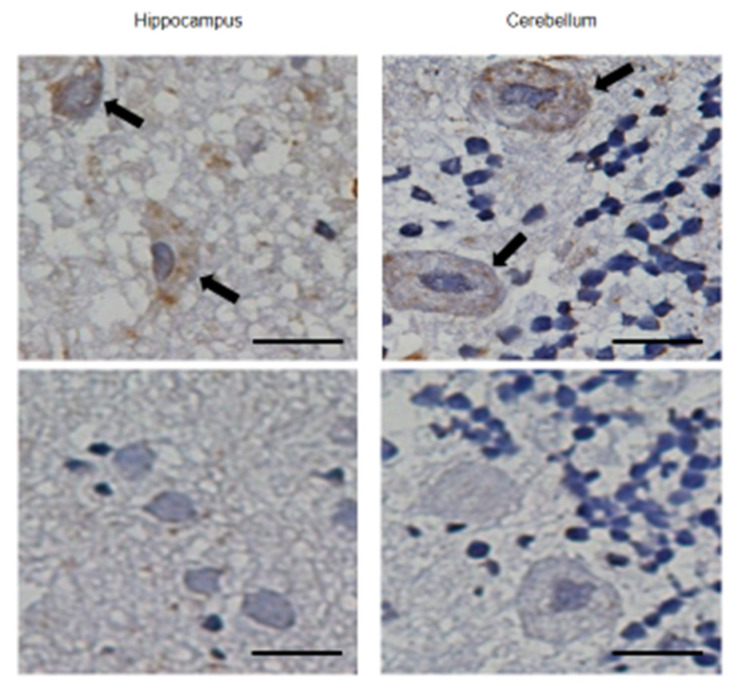
Delivery of pabinafusp alfa to neuronal cells in the brain of a cynomolgus monkey. Pabinafusp alfa was intravenously administered to cynomolgus monkey at a dose of 5 mg/kg, and their brains were resected at 8 h after the administration. Arrows in upper panels indicate pyramidal cells in the hippocampus (**left**) and Purkinje cells in the cerebellum (**right**). Lower panels show negative control (administered control was IgG, which does not cross the BBB). Scale bars, 20 mm. Updated from [11].

The major advantage of pabinafusp alfa is the fusion of anti-hTfR antibodies to IDS, which gives it the ability to pass through the BBB to reach CNS tissues [16,17]. Figure 1 shows the results of an immunohistochemical analysis in which pabinafusp alfa, when administered intravenously to cynomolgus monkeys and hTfR-expressing mice, was found in neuronal cells in different brain regions [11]. These results indicate that, thanks to its TfR-binding ability, pabinafusp alfa reaches the brain parenchyma by crossing the BBB via TfR-mediated transcytosis.

#### 2.1.2. Substrate Reduction

**Figure 2:**
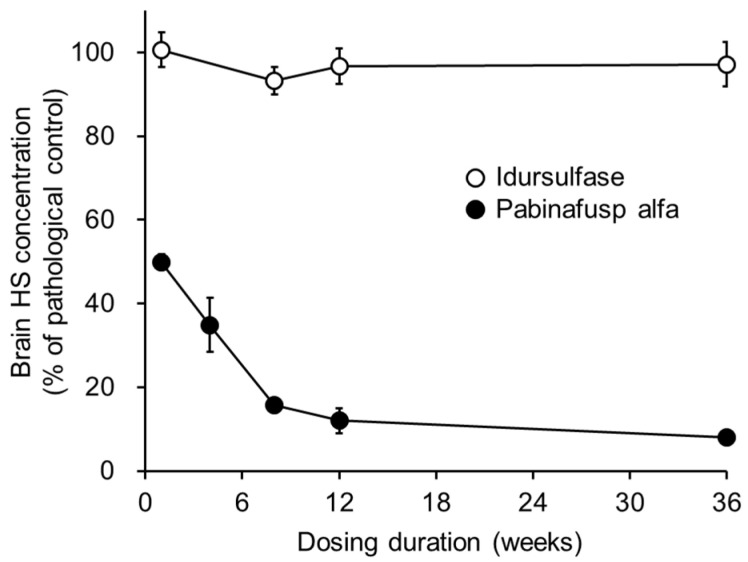
Substrate reducing efficacy of pabinafusp alfa in the brain of MPS II mice. Pabinafusp alfa was intravenously administered to mice at a dose of 2 mg/kg once per week for 1, 4, 8, 12, or 36 weeks. The dose of idursulfase was 0.5 mg/kg/week. Data are from independent experiments (mean with S.D. bars, *n* = 3–5).

The primary pathogenesis of MPS II is the systemic intracellular accumulation of GAGs due to an inherited dysfunction or deficiency of IDS [18,19]. Therefore, the substrate-reducing activity of pabinafusp alfa was evaluated as an indicator of its pharmacological efficacy in an MPS II mouse model using IDS-deficient mice expressing hTfR [11]. The repeated intravenous administration of pabinafusp alfa dose-dependently reduced the accumulation of GAGs (i.e., HS and dermatan sulfate (DS)) in the tissues and organs, including the brain [20]. The HS concentration in the brain rapidly decreased before a maximum reduction was achieved at around 10 weeks of dosing and continued to decrease moderately thereafter (Figure 2). These results clearly indicate that intravenously administered pabinafusp alfa is efficacious against the pathogenic accumulation of GAGs, not only in the peripheral tissues and organs but also in the brain. This is in sharp contrast to conventional intravenous ERT with idursulfase, which does not affect the HS concentration in the brain at all (Figure 2).

#### 2.1.3. Prevention of Neuroinflammation and Subsequent Neurodegeneration

**Figure 3:**
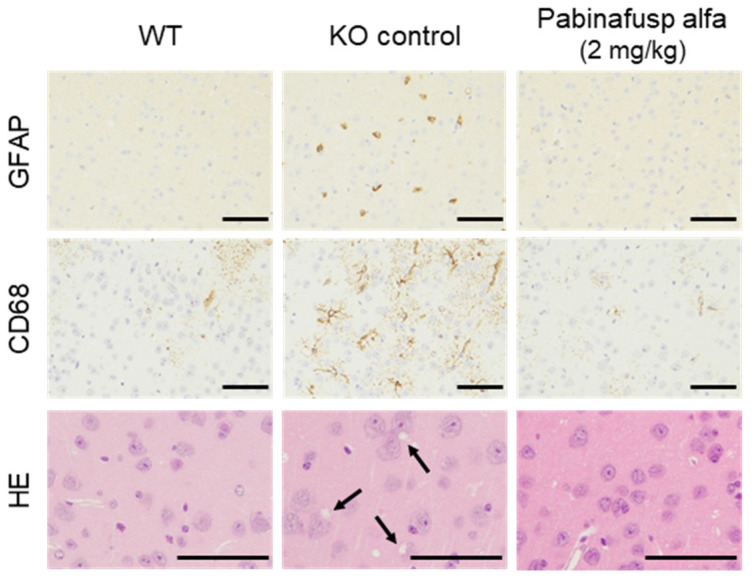
Prevention of neuroinflammation and neurodegeneration by pabinafusp alfa in MPS II mouse brains. Specimens from the brain cortex were stained with GFAP (**top**), CD68 (**middle**), and hematoxylin/eosin (**bottom**). Data are from a 36-week study. Arrows indicate the vacuolation of neuronal cells. Scale bars, 50μm. Updated from [20].

The neurodegenerative processes in MPS II mice were preceded by the activation of glial cells [21,22]. For instance, the intensity of glial fibrillary acidic protein (GFAP) signals increased in the astroglia, as did the number of CD68-positive microglia in the brain cortex (Figure 3). These histopathological changes were suppressed by chronic intravenous treatment with pabinafusp alfa (Figure 3). The relief from neuroinflammation afforded by pabinafusp alfa further prevented morphological abnormalities and neuronal death in the brains of untreated MPS II mice (Figure 3), whereas idursulfase was found to be ineffective against these pathological changes in the brain [20].

#### 2.1.4. Prevention of Neurocognitive Abnormalities

**Figure 4:**
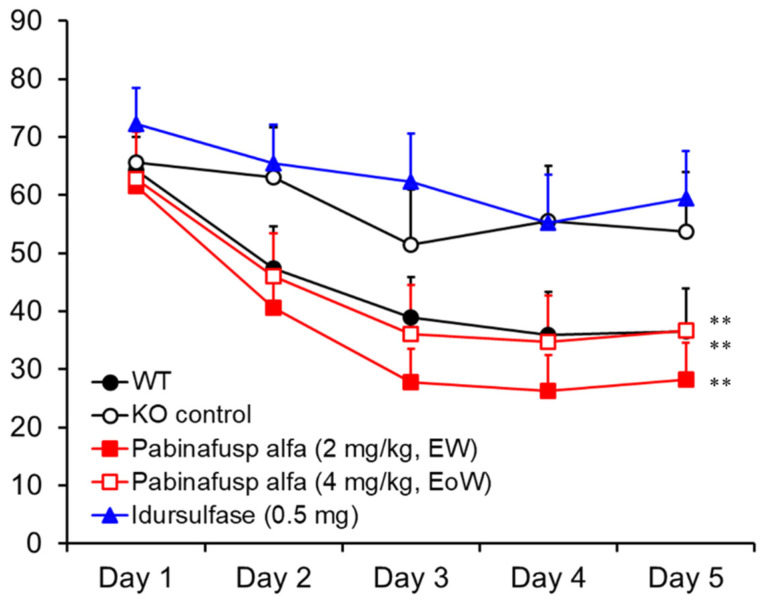
Maintenance of spatial learning abilities in MPS II mice receiving chronic treatment with pabinafusp alfa. After 36 weeks of treatment, spatial learning ability was assessed with the Morris water maze test. The time to reach the platform (goal latency) was measured 3 times per day and the means were calculated within each day for individual animals. Values are presented as the mean with S.E. for each group (*n* = 12–15). Paired *t*-test, ** *p* < 0.01 (Day 1 vs. Day 5). EW, every week; EoW, every other week. Reproduced from [20].

In MPS II mice, progressive neurocognitive impairments manifest themselves as a loss of spatial learning ability, which can be assessed using the Morris water maze test [23]. When normal healthy mice were subjected to the test, the time taken to reach the platform (goal latency) became shorter day by day, whereas untreated MPS II mice experienced difficulty in learning how to reach the platform [20] (Figure 4). The mice receiving chronic treatment with pabinafusp alfa maintained their spatial learning ability, unlike the wild-type animals (Figure 4). Idursulfase failed to prevent the loss of learning ability, so the attenuation of neurocognitive abnormalities observed in the pabinafusp alfa-treated MPS II mice can be primarily attributed to the clearance of HS deposited in the brain. In other words, a high HS concentration in the brain can be viewed as a good predictor of neurodegeneration as well as a marker of drug efficacy in patients with neuronopathic MPS II.

#### 2.1.5. Identification of biomarker for CNS efficacy

**Figure 5:**
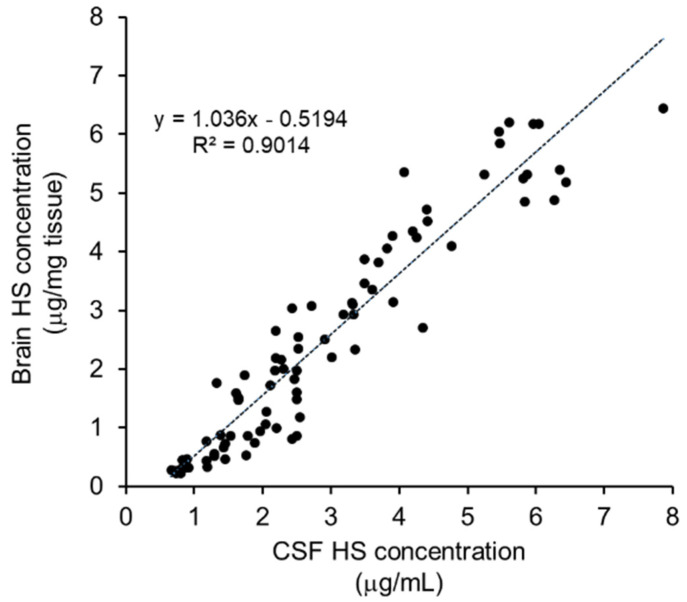
Correlation between concentrations of HS in the brains and the CSF of MPS II mice treated with pabinafusp alfa. Results from studies of single-dose, 4-week, 8-week, 12-week, and 36-week treatments are included.

Thanks to the weakness of the barrier between the brain parenchyma and the cerebrospinal fluid (CSF) [24], HS concentrations in the brain are considered to be directly correlated with those in the CSF, as demonstrated by the high correlations we found between the intracerebral and CSF HS concentrations in the MPS II mice treated with pabinafusp alfa (Figure 5). Thus, HS concentrations in the CSF are a useful and practical surrogate biomarker for monitoring drug efficacy in patients with neuronopathic MPS II, because HS concentrations in the brain cannot be measured in clinical settings.

### 2.2. Preclinical Safety Results

In vitro assay systems were used to comprehensively evaluate the preclinical safety of pabinafusp alfa in cynomolgus monkeys. Since pabinafusp alfa contains an entire IgG structure in its molecule, its safety evaluation needs to involve antibody-associated functions, such as effector functions relevant to cytotoxicity [25]. In this regard, the potential effects of pabinafusp alfa on antibody-dependent cellular cytotoxicity (ADCC) and complement-dependent cytotoxicity (CDC) were examined with TfR-expressing hematopoietic cells, which elicited neither ADCC nor CDC [26]. Consistent with these findings, chronic treatment with the drug did not cause anemia in cynomolgus monkeys. In addition, the binding of pabinafusp alfa with TfR did not interfere with transferrin–TfR interaction. Repeat-dose toxicity studies in cynomolgus monkeys showed no significant toxicological changes at weekly doses of up to 30 mg/kg of pabinafusp alfa, without affecting the iron metabolism. Overall, the preclinical safety studies suggested no significant safey concerns that could be considered clinically relevant to patients with MPS II.

### 2.3. Clinical Results

#### 2.3.1. Clinical Efficacy Data

##### Substrate Reduction in the CSF

**Figure 6:**
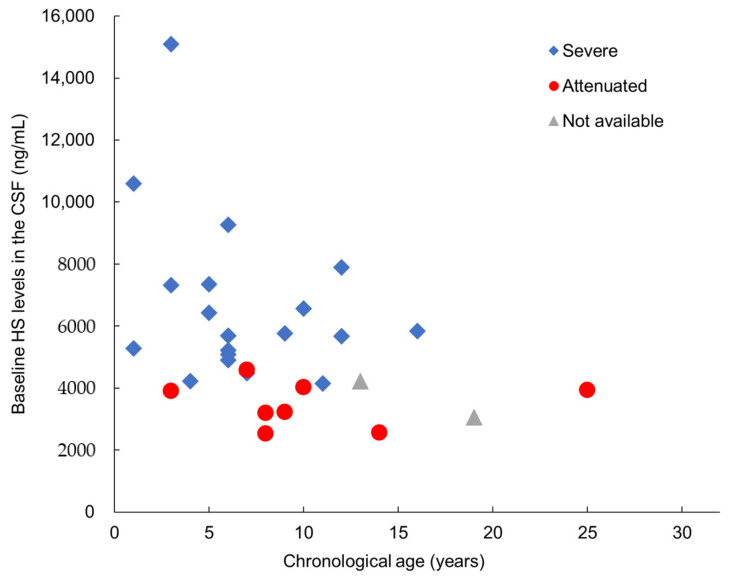
Baseline HS concentrations in the CSF of the 29 patients in the phase II/III clinical trial of pabinafusp alfa (modified from [27]).

On the basis of the preclinical findings (Section 2.1.4), the HS levels in the CSF were stipulated as the primary efficacy surrogate endpoint in the three clinical trials conducted so far. Figure 6 shows the baseline HS levels in the CSF of the 29 patients in the phase I/II and III studies in Japan, which correlated with the disease severity ascribed to each patient by physicians based on their clinical judgment. The HS level in the CSF serves as an accurate indicator of neurodegenerative severity as well as a predictor of clinical outcomes in terms of CNS manifestations. Most patients with attenuated subtypes show HS levels below 4000 ng/mL, which may well indicate that this level is the threshold below which CNS manifestations seldom, or only very slowly, develop. Therefore, reduction or maintenance of HS levels may be useful as a tentative treatment goal for ERT.

**Figure 7:**
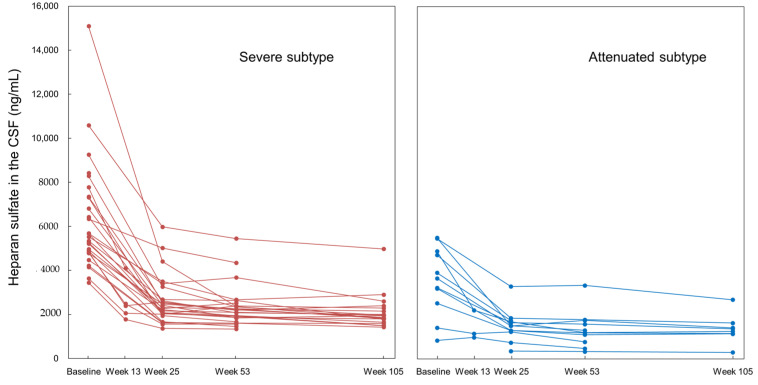
Reductions in the HS concentrations in the CSF of patients with severe and attenuated subtypes of MPS II in phase II and II/III studies.

Figure 7 shows the changes in the HS levels in the CSF of all 39 patients in the phase II/III study in Japan, the phase II study in Brazil, and the respective extension studies. The data are presented with respect to their MPS subtypes (severe or attenuated) and all patients received pabinafusp alfa for 104 weeks at either 2.0 or 4.0mg/kg. In the 2.0 mg/kg group in the phase II and phase II/III studies, the HS concentrations in the CSF significantly decreased between week 1 and week 26 (*p* < 0.001), with a difference of −3366 ± 1923 ng/mL (relative changes from week 1 to week 26: −57.655 ± 11.500%). HS concentrations in the CSF decreased in all subjects in both groups. Notably, the treatment reduced the CSF HS levels in the majority of the patients to below the threshold level of 4000 ng/m.

##### Neurocognitive Efficacy

In the studies carried out in Japan, neurocognitive development was evaluated according to the Kyoto Scale of Psychological Development (KSPD). This corresponds to the Bayley scales of infant and toddler development, third edition (BSID-III), which was employed for patients in Brazil with developmental ages younger than 42 months (age-equivalent (AE) scores), while the Kaufman Assessment Battery for Children, 2nd edition (KABCII), was used for older patients.

**Figure 8:**
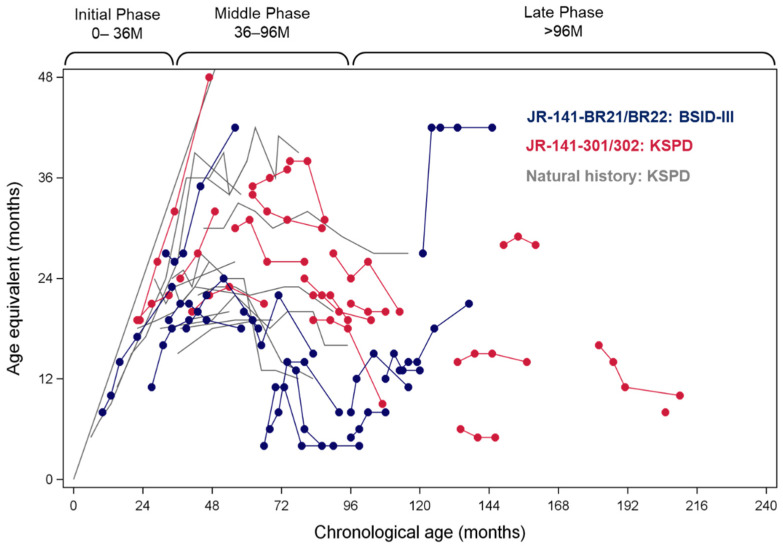
Age equivalent scores in the phase II and phase II/III studies in patients with the severe subtype of MPS-II, overlaid onto the corresponding developmental trajectories from the natural history data. JR-141-BR21/BR22 stands for the phase II study in Brazil and its extension study; JR-141-301/302 indicates the phase II/III study in Japan and its extension study (updated from [14,15]).

Figure 8 shows the changes in AE scores in the patients with the severe subtype of MPS-II in the phase II and II/III studies, overlaid onto those from the natural history data of Japanese patients with the severe subtype [28]. Almost all of the patients in Brazil showed a marked improvement in AE scores over 104 weeks, while most of the Japanese patients showed stabilization, along with improvement in some.

Table 1 numerically substantiates these AE score changes in patients with both the attenuated and severe subtypes. At week 52, improvement or stabilization in terms of AE score changes were observed in 85% of patients with the two subtypes in Japan, as well as 94% of those in Brazil. At week 104, 62% of the patients in Japan and 75% of those in Brazil showed improvement or stabilization. Taken together, these results show that pabinafusp alfa brought about the improvement or stabilization of neurocognitive impairment in 89% of patients with MPS-II irrespective of their subtypes at week 52, and in 75% of them at week 104.

In addition to the standardized neurocognitive assessments, clinical behavioral observations by the subjects’ families and investigators were collected in order to register subtle but potentially meaningful behavioral changes that the standardized assessments might fail to capture, in particular in patients with the advanced severe subtype (detailed tabulated reports of the narrative records are published elsewhere [14,15]).

In both the phase II/III and II studies, similar behavioral changes were observed across three major areas (speech, motor functions, and liveliness/expression). Positive behavioral changes in speech included increased rates of utterances, better verbal responsiveness, and the resumption of singing. In terms of liveliness/expression, stable mood, less agitation, and more smiling were often reported. These positive changes were observed across all ages and subtypes, although the younger subjects showed more marked improvements than the older ones. Notably, even among the subjects without marked improvements in their speech or motor functions (especially adult subjects with a long history of disease), positive changes in important attachment behaviors (e.g., smiling) [29] were still recognizable.

The fact that these subjective, non-standardized observations are in accordance with the objective findings from the neurodevelopmental scales seems to further indicate the neurocognitive efficacy of pabinafusp alfa across all patient populations of different ages and subtypes.

##### Somatic Efficacy

**Figure 9:**
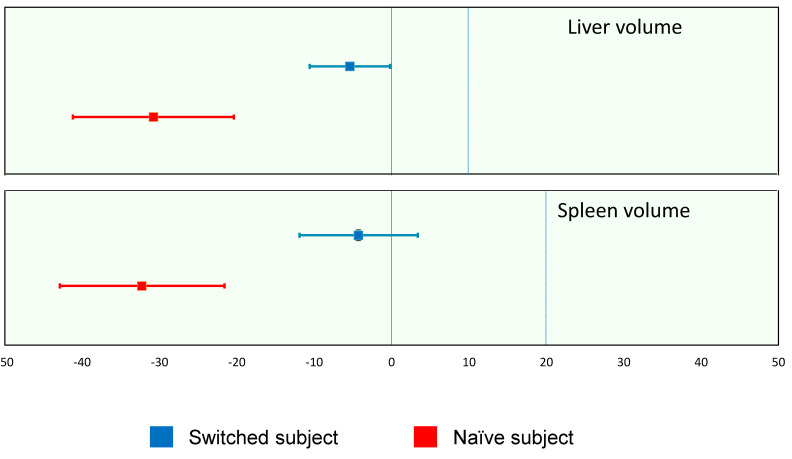
Relative changes in the liver and spleen volumes from baseline to week 52. The blue dotted lines are suggested thresholds of clinically significant changes (10% for liver volume and 20% for spleen volume); values below these can be interpreted as either the stabilization or improvement of hepatosplenomegaly (updated from [14,15]).

As a measure of the efficacy of pabinafusp alfa against the somatic symptoms of MPS II, liver and spleen volumes are reported here as representative efficacy endpoints, as progressive hepatosplenomegaly is a prominent clinical feature of the disease. In evaluating the changes in organomegaly, attention needs to be paid to the large variability in organ volumes in pediatric subjects; thus, individual subjects’ relative volume changes were recorded at week 52, in comparison with the baseline volume defined as 100%. The liver and spleen volumes significantly decreased in the naïve patients without prior ERT, and they also decreased by about 5% in patients who were switched from conventional ERT to pabinafusp alfa (Table 2, Figure 9).

These results, along with other somatic efficacy data (e.g., changes in serum HS and DS concentrations and cardiac function [14,15]), suggest that the efficacy of pabinafusp alfa against somatic symptoms is comparable to that of conventional idursulfase.

#### 2.3.2. Clinical Safety Data

The safety of pabinafusp alfa was evaluated on the basis of the results of the phase I/II, II, and II/III studies, and safety was confirmed in patients in the 1.0, 2.0, and 4.0 mg/kg groups. The optimal weekly dose of pabinafusp alfa was considered to be 2.0 mg/kg, because most of the adverse drug reactions (ADRs) in this group were mild and all were duly managed without patients having to withdraw from the study. A summary of the clinical safety of pabinafusp alfa is shown in Table 3. It is of note that no dose-limiting toxicities were observed at 4.0 mg/kg in the phase II study in Brazil, even though the infusion-associated reactiosn (IARs) were observed most frequently at this dose.

Eleven non-fatal serious adverse events took place in the three studies, none of which were related to pabinafusp alfa. Both of the two reported deaths were due to respiratory failure as an exacerbation of MPS-II and were unrelated to the drug. In summary, pabinafasup alfa was found to be generally well tolerated at doses of up to 4 mg/kg, and its long-term safety up to 104 weeks has been confirmed as acceptable.

## 3. Discussion

To date, pabinafusp alfa is the first and only recombinant enzyme that can be successfully delivered via intravenous administration across the BBB. This article presents the latest preclinical data as well as the longest clinical data hitherto accumulated (up to 104 weeks), which demonstrate the clinically meaningful efficacy of this treatment against the CNS symptoms of MPS-II, whilst also showing its efficacy against somatic symptoms.

The establishment of this novel IV ERT with both central and peripheral efficacy has overcome formidable challenges. Despite the fairly straightforward basic pathophysiology of neuronopathic MPS, which starts as a genetic enzyme deficiency that leads to the accumulation of uncatabolized substrates in the CNS and progressive neurodegeneration, details of the functional and structural neuronal damages it causes remain to be elucidated [3]. In order to unravel the complexities behind this pathogenesis and progression, we used a three-pronged approach. First, we evaluated the initial component of the pathogenesis (i.e., substrate accumulation) by measuring HS concentrations in the brain and the CSF. Second, we carried out histopathological evaluations to investigate the neurodegeneration, which was the second component. Third, we examined behavioral abnormalities representing CNS manifestations, thereby capturing the final component of these neurodegenerative events.

The systemic GAG accumulations seen in our MPS II mouse model were reduced dose-dependently by the intravenous administration of pabinafusp alfa (Section 2.1.2), which then duly suppressed neuroinflammation and other neuropathological abnormalities (Section 2.1.3), leading to the normalization of impaired spatial learning abilities (Section 2.1.4). These preclinical findings corroborated the efficacy of pabinafusp alfa through all three components of the pathogenesis of neuronopathy and encouraged the translation of these findings into clinical studies.

**Figure 10:**
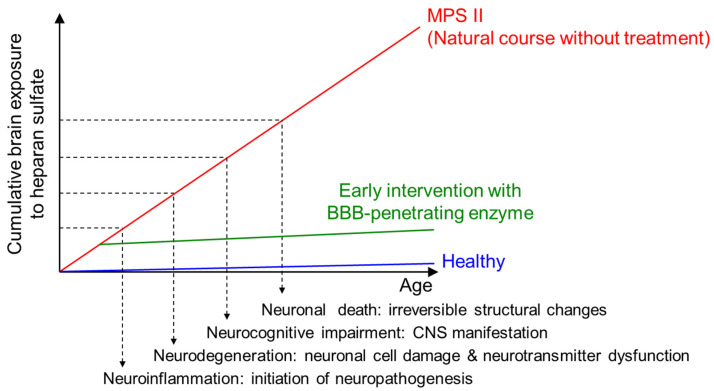
Conceptual association between cumulative exposure of the brain to HS and CNS disease progression in patients with neuronopathic MPS II. The vertical dashed arrows indicate the presumed timings of the onset of neuronopathic events. The Y-intercepts with dashed lines indicate the postulated levels of cumulative exposure of the brain to HS when a corresponding neuropathology evolves. The red and blue lines indicate the cumulative exposures to HS in patients with MPS II and healthy subjects, respectively. Early intervention with pabinafusp alfa decreases the intracerebral HS concentrations and thereby reduces the cumulative exposure, preventing or delaying the onset of CNS involvement. Even if the treatment starts when some symptoms have already developed, the clearance of HS accumulations in the brain may be able to reverse the symptoms by correcting neuronal dysfunctions before irreversible (mostly structural) CNS damage occurs (updated from [21]).

We found that the extent of damage to the CNS and its manifestations in MPS II mice were not solely determined by the HS concentrations in the brain but also by the duration of HS elevation [21], suggesting the cumulative pathogenicity of intracerebral HS accumulation. It was clear, therefore, that temporal factors must be taken into account to better address the onset and progression of neurodegeneration (Figure 10) [21]. This underpins the importance of the early introduction of ERT for patients with neuronopathic MPS-II so that the period of elevated HS levels in the brain can be shortened to prevent or ameliorate neurocognitive impairment in the future. Indeed, our 104-week neurodevelopmental data show almost normal developmental trajectories in some of the very young patients given pabinafusp alfa, unlike in the older patients (Figure 8). This point is poignantly exemplified by the markedly divergent developmental trajectories of two siblings with MPS-II, one treated with conventional ERT and the other treated from early on with pabinafusp alfa [29].

One methodological limitation in the clinical efficacy evaluation of the three clinical trials relates to the neurocognitive data collection in Japan and Brazil using different scales—KSPD in the former and BSID-III / KABCII in the latter—for operational reasons. Although these methods are known to correspond to each other, a single, universally validated rating scale would have enabled the more robust integrated analysis and comparison of these developmental data, as the apparently more marked neurocognitive improvement seen in the Brazilian data compared to that in the Japanese data is difficult to interpret due to the different scales employed.

The development of novel therapeutics for a rare disease invariably involves a dilemma: balancing the difficulty of evaluating the drug’s efficacy against a disease that is perhaps not fully understood and with few patients available for clinical trials on the one hand with the need to expedite development to meet urgent medical needs on the other. MPS-II is known to be a heterogeneous yet progressive, debilitating, and often fatal disease. While long-term functional and structural assessments would have provided more robust efficacy data, a realistic compromise had to be made to advance the development of pabinafusp alfa by capturing both the biochemical surrogate endpoints and the clinical endpoints reflecting CNS manifestations. In other words, this study examined both the initial process of neurodegeneration and, at the same time, some of the clinical neuropsychiatric manifestations as the final outcome of the long and complex pathological process. Limitations in the reported clinical studies, in particular with respect to the long-term neurodevelopmental data, need to be addressed in post-marketing studies in Japan and the planned phase III global trial, which will provide further evidence of the dual efficacy of pabinafusp alfa against both somatic and CNS symptoms in patients with MPS-II.

## 4. Materials and Methods

### 4.1. Preclinical Studies

#### 4.1.1. Animals

hTfR-KI/Ids-KO mice, a mouse model of MPS II, were used as described previously [11], and C57BL/6 mice (Charles River, Yokohama, Japan) were used as a normal control. The cynomolgus monkeys (*Macaca fascicularis*) used had all been purpose-bred for research (Shin Nippon Biomedical Laboratories, Kagoshima, Japan). All animal experiments were conducted under the approval of the Animal Care and Use Committees of Shin Nippon Biomedical Laboratories (IACUC250-155, 30 June 2016; IACUC250-166, 13 September 2016; IACUC250-183, 6 June 2017), and JCR Pharmaceuticals (JR141-P1803, 27 March 2018).

#### 4.1.2. BBB Penetration

The brain delivery of pabinafusp alfa by BBB penetration was determined by immunohistochemical analysis in cynomolgus monkeys that had received intravenous infusion of the drug at a level of 5 mg/kg. Pabinafusp alfa was detected with an HRP-labeled human IgG antibody. Detailed methods are described elsewhere [11].

#### 4.1.3. Substrate Reduction

To evaluate the efficacy of pabinafusp alfa in reducing substrate accumulations, the drug was administered to MPS II mice through the tail vein at a level of 2 mg/kg once a week for 1, 4, 8, 12, or 36 weeks. Control mice were given idursulfase at a level of 0.5 mg/kg once a week. One week after the final dosing, tissues and organs, including the brain and CSF, were collected so that the HS concentrations could be measured by liquid chromatography-tandem mass spectrometry [12].

#### 4.1.4. Evaluation of Neuroinflammation and Neurodegeneration

Pabinafusp alfa was intravenously administered to MPS II mice at a level of 2 mg/kg once a week for 36 weeks. One week after the final dosing, tissues and organs were collected and brains were subjected to histopathological analysis [21]. The expression of GFAP was used as a marker for the activation of the astrocytes, while the expression of CD68 was used as a marker for the activation of microglial cells. Staining with hematoxylin and eosin was performed to detect morphological changes in neuronal cells.

#### 4.1.5. Evaluation of Neurocognitive Abnormalities

To examine spatial learning ability, the Morris water maze test was performed after 36 weeks of treatment. Briefly, each mouse was placed with its head facing the wall of a circular pool equipped with a transparent acrylic resin platform, and the time taken to reach the platform (goal latency) was measured [21].

#### 4.1.6. Safety

Safety evaluations using in vitro assays in cynomolgus monkeys were carried out as previously described [26].

### 4.2. Clinial Studies

The study designs, procedures, outcomes, statistical analyses, and other details of the three clinical trials of pabinafusp alfa are summarized in Table S1. IRB approval details for the trials in Japan and Brazil are summarized in Tables S2–S4.

## 5. Conclusions

This article summarizes and updates our preclinical and clinical evidence of the dual efficacy of pabinafusp alfa against both the central and peripheral/somatic symptoms of neuronopathic MPS-II. The drug’s mechanism of action has been highlighted: intravenously administered IDS is delivered to the body via M6P- and TfR-mediated transcytoses and into the brain parenchyma via TfR-mediated transcytosis. By reducing HS accumulation in the brain, pabinafusp alfa prevents or alleviates neurodegeneration. Further long-term data on more patients are expected to provide more evidence of the benefits of this novel drug, and we hope that its mechanism of action can, in due course, be applied to treat other neuronopathic lysosomal storage diseases so that their hitherto unaddressed CNS manifestations can also be better managed.

## Figures and Tables

**Table 1:** Changes in AE scores on KSPD (for Japan), BSID-III, and KABCII (for Brazil). Changes in AE scores exceeding 3 months were defined as improvement, while those ±3 months were defined as stabilization and those below 3 months were defined as deterioration (updated from [14,15]).

Week	Changes in AE scores	Japan			Brazil			Total
		Severe	Attenuated	Total	Severe	Attenuated	Total	
Week 52	Improvement	2 (11%)	8 (100%)	10 (37%)	8 (57%)	5 (100%)	13 (68%)	23 (50%)
	Stabilization	13 (68%)	0	13 (48%)	5 (36%)	0	5 (26%)	18 (39%)
	Deterioration	4 (22%)	0	4 (15%)	1 (7%)	0	1 (5%)	5 (11%)
Week 104	Improvement	4 (27%)	1 (100%)	5 (31%)	6 (50%)	5 (100%)	11 (65%)	16 (48%)
	Stabilization	5 (33%)	0	5 (31%)	4 (33%)	0	4 (24%)	9 (27%)
	Deterioration	6 (40%)	0	6 (38%)	2 (17%)	0	2 (12%)	8 (24%)

**Table 2:** Liver and spleen volumes (updated from [14,15]).

Organs	ERT Status	*n*	Mean (SD)	Median [Min–Max]	95% CI
Liver volume	Switched subjects	28	−5.4 (13.3)	−6.0 [−38.0–23.4]	−10.6–−0.2
	Naïve subjects	4	−30.8 (6.5)	−31.1 [−38.4–−22.6]	−41.2–−20.4
Spleen volume	Switched subjects	28	−4.3 (19.7)	−3.7 [−62.2–31.6]	−11.9–3.4
	Naïve subjects	4	−32.3 (6.7)	−33.3 [−38.7–−23.8]	−42.9–−21.6

**Table 3:** Summary of adverse events and adverse drug reactions for pabinafusp alfa in the phase II/III and II studies (updated from [14,15]).

	Phase I/II Study in Japan			Phase II/III Study in Japan			Phase II Study in Brazil			All		
Adverse Events/Reactions	*n*	Proportion (%)	Number of Events	*n*	Proportion (%)	Number of Events	*n*	Proportion (%)	Number of Events	*n*	Proportion (%)	Number of Events
Number of subjects	14	--	--	28	--	--	20	--	--	62	--	--
Adverse events	9	64.3	20	28	100.0	340	20	100.0	202	57	91.9	562
Serious adverse events	1	7.1	1	5	17.9	10	7	35.0	7	13	21.0	18
(Deaths)	0	0.0	0	1	3.6	2	1	5.0	1	2	3.2	3
Significant adverse events	4	28.6	8	17	60.7	61	11	55.0	47	32	51.6	116
(Infusion associated reaction)	4	28.6	8	14	50.0	51	10	50.0	45	28	45.2	104
Adverse drug reactions	7	50.0	11	15	53.6	59	11	55.0	46	33	53.2	116
Serious adverse drug reactions	1	7.1	1	0	0.0	0	0	0.0	0	1	1.6	1
(Deaths)	0	0.0	0	0	0.0	0	0	0.0	0	0	0.0	0
Significant adverse drug reactions	4	28.6	8	14	50.0	51	10	50.0	45	28	45.2	104
(Infusion associated reaction)	4	28.6	8	14	50.0	51	10	50.0	45	28	45.2	104

## Data Availability

The data presented in this study may be available on request from the corresponding author. The data are not publicly available due to the intellectual property rights for pabinafusp alfa.

## Supplementary Materials

The following are available online at https://www.mdpi.com/article/10.3390/ijms222010938/s1, Table S1: details of the clinical trials with pabinafusp alfa; Tables S2–S4: IRB approval details for the three clinical trials in Japan and Brazil.

## References

[1] Bigger B.W., Begley D.J., Virgintino D., Pshezhetsky A.V. (2018). Anatomical changes and pathophysiology of the brain in mucopolysaccharidosis disorders. Mol. Genet. Metab..

[2] Jakobkiewicz-Banecka J., Gabig-Ciminska M., Kloska A., Malinowska M., Piotrowska E., Banecka-Majkutewicz Z., Banecki B., Wegrzyn A., Wegrzyn G. (2016). Glycosaminoglycans and mucopolysaccharidosis type III. Front. Biosci..

[3] Sato Y., Okuyama T. (2020). Novel Enzyme Replacement Therapies for Neuropathic Mucopolysaccharidoses. Int. J. Mol. Sci..

[4] Burton B.K., Jego V., Mikl J., Jones S.A. (2017). Survival in idursulfase-treated and untreated patients with mucopolysaccharidosis type II: Data from the Hunter Outcome Survey (HOS). J. Inherit. Metab. Dis..

[5] Muenzer J., Hendriksz C.J., Fan Z., Vijayaraghavan S., Perry V., Santra S., Solanki G.A., Mascelli M.A., Pan L., Wang N. (2016). A phase I/II study of intrathecal idursulfase-IT in children with severe mucopolysaccharidosis II. Genet. Med..

[6] Eisengart J.B., Pierpont E.I., Kaizer A.M., Rudser K.D., King K.E., Pasquali M., Polgree L.E., Dickson P.I., Le S.Q., Miller W.P. (2019). Intrathecal enzyme replacement for Hurler syndrome: Biomarker association with neurocognitive outcomes. Genet. Med..

[7] Seo J.H., Kosuga M., Hamazaki T., Shintaku H., Okuyama T. (2021). Impact of intracerebroventricular enzyme replacement therapy in patients with neuronopathic mucopolysaccharidosis type II. Mol. Ther. Methods Clin. Dev..

[8] Boado R.J., Hui E.K.-W., Lu J.Z., Pardridge W.M. (2014). Insulin receptor antibody-iduronate 2-sulfatase fusion protein: Pharmacokinetics, anti-drug antibody, and safety pharmacology in Rhesus monkeys. Biotechnol. Bioeng..

[9] Giugliani R., Giugliani L., de Oliveira Poswar F., Donis K.C., Corte A.D., Schmidt M., Boado R.J., Nestrasil I., Nguyen C., Chen S. (2018). Neurocognitive and somatic stabilization in pediatric patients with severe Mucopolysaccharidosis Type I after 52 weeks of intravenous brain-penetrating insulin receptor antibody-iduronidase fusion protein (valanafusp alpha): An open label phase 1-2 trial. Orphanet. J. Rare Dis..

[10] Couch J.A., Yu Y.J., Zhang Y., Tarrant J.M., Fuji R.N., Meilandt W.J., Solanoy H., Tong R.K., Hoyte K., Luk W. (2013). Addressing safety liabilities of TfR bispecific antibodies that cross the blood-brain barrier. Sci. Transl. Med..

[11] Sonoda H., Morimoto H., Yoden E., Koshimura Y., Kinoshita M., Golovina G., Takagi H., Yamamoto R., Minami K., Mizoguchi A. (2018). A Blood-Brain-Barrier-Penetrating Anti-human Transferrin Receptor Antibody Fusion Protein for Neuronopathic Mucopolysaccharidosis II. Mol. Ther..

[12] Tanaka N., Kida S., Kinoshita M., Morimoto H., Shibasaki T., Tachibana K., Yamamoto R. (2018). Evaluation of cerebrospinal fluid heparan sulfate as a biomarker of neuropathology in a murine model of mucopolysaccharidosis type II using high-sensitivity LC/MS/MS. Mol. Genet. Metab..

[13] Okuyama T., Eto Y., Sakai N., Minami K., Yamamoto T., Sonoda H., Yamaoka M., Tachibana K., Hirato T., Sato Y. (2019). Iduronate-2-Sulfatase with Antihuman Transferrin Receptor Antibody for Neuropathic Mucopolysaccharidosis II: A Phase 1/2 Trial. Mol. Ther..

[14] Giugliani R., Martins A.M., So S., Yamamoto T., Yamaoka M., Ikeda T., Tanizawa K., Sonoda H., Schmidt M., Sato Y. (2021). Iduronate-2-sulfatase fused with anti-hTfR antibody, pabinafusp alfa, for MPS-II: A phase 2 trial in Brazil. Mol. Ther..

[15] Okuyama T., Eto Y., Sakai N., Nakamura K., Yamamoto T., Yamaoka M., Ikeda T., So S., Tanizawa K., Sonoda H. (2021). A phase 2/3 trial of pabinafusp alfa, IDS fused with anti-human transferrin receptor antibody, targeting neurodegeneration in MPS-II. Mol. Ther..

[16] Pardridge W.M. (1986). Receptor-mediated peptide transport through the bloodbrain barrier. Endocr. Rev..

[17] Pardridge W.M., Eisenberg J., Yang J. (1987). Human blood-brain barrier transferrin receptor. Metabolism.

[18] Neufeld E.F., Muenzer J., Scriver C.R., Beaudet A.L., Sly W.S., Valle D. (2001). The Mucopolysaccharidoses. The Metabolic & Molecular Bases of Inherited Disease.

[19] Tylki-Szymanska A. (2014). Mucopolysaccharidosis type II, Hunter’s syndrome. Pediatr. Endocrinol. Rev..

[20] Morimoto H., Kida S., Yoden E., Kinoshita M., Tanaka N., Yamamoto R., Koshimura Y., Takagi H., Takahashi K., Hirato T. (2021). Clearance of heparan sulfate in the brain prevents neurodegeneration and neurocognitive impairment in MPS II mice. Mol. Ther..

[21] Fusar Poli E., Zalfa C., D’Avanzo F., Tomanin R., Carlessi L., Bossi M., Nodari L.R., Binda E., Marmiroli P., Scarpa M. (2013). Murine neural stem cells model Hunter disease in vitro: Glial cell-mediated neurodegeneration as a possible mechanism involved. Cell Death Dis..

[22] Zalfa C., Verpelli C., D’Avanzo F., Tomanin R., Vicidomini C., Cajola L., Manara R., Sala C., Scarpa M., Vescovi A.L. (2016). Glial degeneration with oxidative damage drives neuronal demise in MPSII disease. Cell Death Dis..

[23] D’Hooge R., De Deyn P.P. (2001). Applications of the Morris water maze in the study of learning and memory. Brain Res. Rev..

[24] Jiménez A.J., Domínguez-Pinos M.D., Guerra M.M., Fernández-Llebrez P., Pérez-Fígares J.M. (2014). Structure and function of the ependymal barrier and diseases associated with ependyma disruption. Tissue Barriers.

[25] Vidarsson G., Dekkers G., Rispens T. (2014). IgG subclasses and allotypes: From structure to effector functions. Front. Immunol..

[26] Yamanoto R., Yoden E., Tanaka N., Kinoshita M., Imakiire A., Hirato T., Minami K. (2021). Nonclinical safety evaluation of pabinafusp alfa, an anti-human transferrin receptor antibody and iduronate-2-sulfatase fusion protein, for the treatment of neuronopathic mucopolysaccharidosis type II. Mol. Genet. Metab. Rep..

[27] Tomita K., Okamoto S., Seto T., Hamazaki T., So S., Yamamoto T., Tanizawa K., Sonoda H., Sato Y. (2021). Divergent developmental trajectories in two siblings with neuropathic mucopolysaccharidosis type II (Hunter syndrome) receiving conventional and novel enzyme replacement therapies: A case report. JIMD Rep..

[28] Seo J.-H., Okuyama T., Shapiro E., Fukuhara Y., Kosuga M. (2020). Natural history of cognitive development in neuronopathic mucopolysaccharidosis type II (Hunter syndrome): Contribution of genotype to cognitive developmental course. Mol. Gen. Metab. Rep..

[29] Bowlby J. (1969). Attachment.

