# Supplementary material for: Enzyme Replacement Therapy with Pabinafusp Alfa for Neuronopathic Mucopolysaccharidosis II: An Integrated Analysis of Preclinical and Clinical Data
Source: Int J Mol Sci. 2021 Oct 10;22(20):10938. doi: 10.3390/ijms222010938 (PMC8535651; doi:10.3390/ijms222010938)
Supplement: Supplementary file 1 [file ijms-22-10938-s001.zip › ijms-1373741-supplementary.pdf]

Table S1. Details of the clinical trials with pabinafusp alfa

Study	Phase I/II	Phase II/III	Phase II
Study design	-A multicenter, open-label, randomized clinical trial of intravenous pabinafusp alfa(PFA) in patients with MPS II<br>-Conducted in 8 hospitals<br>-Objectives: to assess the safety, pharmacokinetics (PK) and exploratory efficacy of intravenously administered PFA.<br>-Safety was evaluated during the first 4-week part in 2 patients at 0.01, 0.1, 1.0, 2.0 mg/kg/week of PFA<br>-The second 4-week part was a randomized study, during which the safety, PK, and exploratory efficacy of PFA was evaluated at 1.0 or 2.0 mg/kg/week (6 patients in each group)	-A multicenter, open-label clinical trial<br>-Objective: to evaluate the efficacy of intravenous PFA in patients with MPS II<br>-Conducted in 19 hospitals in Japan<br>-The study consisted of a treatment period of 52 weeks, which was preceded by an observation period of 4 weeks for the patients who switched to PFA from previous enzyme replacement therapy (ERT) with idursulfase, or of 2 weeks for those who had received no prior ERT<br>-No wash-out period for the preceding ERT was required before the study.	-An open-label, parallel group, randomized clinical trial<br>-Objectives: to evaluated the safety, PK and efficacy of intravenous PFA in patients with MPS-II<br>-Conducted in 2 hospitals in Brazil<br>-The study consisted of 4 stages:<br>1) screening and confirmation of eligibility<br>2) baseline studies<br>3) randomization and washout<br>4) treatment and assessment<br>-The patients who had been receiving idursulfase underwent a 1-week washout period before switching to PFA

	-In all patients, idursulfase was switched to PFA without a washout period		
Ethical compliance	-The studies complied with the Declaration of Helsinki.<br>-The protocol and procedures regarding informed consent were reviewed and approved by the Institutional Review Board at each participating institution.<br>-All patients or their legal guardians submitted a signed informed consent form prior to enrolment.		
Participants and procedures	A total of 14 male patients were enrolled. Two patients (aged 33 and 63 years) without neurocognitive impairment were selected for the Part 1. In Part 2, the other 12 patients (aged 12.9 ± 5.3 years) were randomly assigned to receive 1.0 or 2.0 mg/kg of PFA.<br>Study protocol：https://clinicaltrials.gov/ct2/show/NCT03128593	A total of 28 male patients received 2.0 mg/kg/week of PFA intravenously. Pretreatment with antihistamines, steroids, and other appropriate medications was allowed during the treatment if infusion-associated reactions were suspected.<br>Study protocol: https://clinicaltrials.gov/ct2/show/NCT03568175).	A total of 20 patients were randomly allocated to 3 PFA dosages: 8 to 1.0 mg/kg, 5 to the 2.0 mg/kg, and 7 to 4.0 mg/kg.<br>Study protocol: https://clinicaltrials.gov/ct2/show/NCT03359213).
Randomization and masking	In Part 2, 11 of the 12 subjects were randomly assigned to receive either 1.0 or a 2.0 mg/kg of PFA at an overall ratio of 1:1.	This was an open-label study with no masking	After screening, the patients were divided into three age groups (0 to 3 years 11 months, 4 to 7 years 11 months, and 8 years or older) and

	The exception was a 6-year-old who was specifically assigned to the 1.0 mg/kg group due to concerns over the potential risks of high-dose administration.<br>This was an open-label study with no masking.		randomly assigned to one of the three dosages, although it was ensured that at least two members of each age group were assigned to each dosage group so that overall randomization to the treatment arms was at a ratio of 1:1:1.
Outcomes	Safety evaluations included monitoring adverse events and laboratory tests (hematologic and serum biochemical tests and urinalyses, heart rate, body temperature, and blood pressure along with electrocardiograms, anti-IDS antibody and anti-PFA antibody levels).<br>PK was evaluated at all dosing points in Part 1, and after the first and last PFA administrations in Part 2.<br>The time points for blood sampling were before dosing, 1 h after the start of dosing,	-The primary efficacy endpoint was the changes in HS concentrations in the CSF at the time of the initial dose and at week 52, which were used an indicators of neurodegeneration. Measurements of HS levels were made with high-sensitivity LC/MS/MS [12]<br>-The secondary efficacy endpoints included developmental assessments with the Kyoto Scale of Psychological Development and Vineland Adaptive Behavior Scales (VABS-II). Investigators' observations of the qualitative behavioral changes in the patients were also collected to register the neuropsychiatric and behavioral changes that are difficult	-Safety evaluations were based on the type and severity of adverse events, vital signs, anti-PFA antibodies, electrocardiography, and routine blood tests (hematology, liver function, renal function, iron-related parameters) and urinalysis.<br>-Efficacy endpoints were (a) changes between baseline and week 26 in serum and urine HS and DS concentrations, liver and spleen volumes by MRI, and left ventricular mass index by echocardiography; and (b) changes between baseline and week 26 in cortical grey matter, ventricular volumes and DTI results

	immediately after the last administration, and then 3, 9, and 21 h afterwards. Drug concentrations were measured by an electrochemiluminescence assay.<br>Evaluations of exploratory efficacy focused on heparin sulfate (HS) and delmatan sulfate (DS) concentrations in the cerebrospinal fluid (CSF). To evaluate somatic efficacy, HS and DS levels in the serum and urine were also measured. Computed tomography (CT) was used to assess hepatosplenomegaly. Cardiac structures and functions were evaluated by echocardiography.	to capture by the standardized methods. To evaluate peripheral efficacy, changes in serum HS and DS concentrations (LC/MS/MS), liver and spleen volumes (CT), cardiac function (echocardiography) were assessed at baseline and at weeks 25 and 52.<br>-Safety endpoints were adverse events, adverse drug reactions, anti-drug antibodies, and infusion-associated reactions, as well as data from laboratory tests and electrocardiography.	by MRI, HS and DS concentrations in the CSF, results of neurocognitive and adaptive behavioral tests (BSID-III, KABC-II and VABS-II), quality of life measurements, and actigraphy readings. LC/MS/MS was used to measure HS and DS concentrations in the CSF, serum, and urine.<br>PK evaluations were performed at all dosing points and after the first and the last administrations of PFA in the patients aged 8 years or older. Blood samples were collected at six time points: 10 minutes prior to infusion, 1 hour after the start of dosing, immediately after the last administration, and then 3, 6, and 21 hours post infusion. PK parameters were measured by electrochemiluminescence assay.
Statistical analysis	A non-compartmental model analysis was used to calculate PK parameters. To analyse PK	The minimum sample size needed to evaluate the primary efficacy endpoint was calculated as in the phase I/II study.	All data analyses followed the intention-to-treat principle. PK analysis was done only for the

	endpoints, plasma drug concentrations adjusted for the plasma IDS concentration at baseline were used.<br>Efficacy was assessed in the full analysis set.<br>Safety was assessed in the safety analysis set, which included all patients who received at least 1 dose of PFA. The number and proportion of subjects who experienced any adverse drug reactions were recorded, along with the total number of adverse drug reactions. Statistical analyses were performed using the SAS version 9.4 statistical software package (SAS Institute Inc., Cary, NC, USA).	The effect size of PFA versus the standard idursulfase treatment in reducing HS concentrations in the CSF was conservatively estimated to be about 1100 ng/mL. Detection of this effect with 80% power using a 2-sided paired t-test at 5% significance level would require five patients for the study. More patients were enrolled to collect sufficient data to evaluate both central and peripheral efficacy.<br>All data analyses followed the intention-to-treat principle.<br>The paired t-test was used to analyze the differences between HS concentrations in the CSF at baseline and at week 52.<br>All statistical analyses were performed with the SAS version 9.4 statistical software package (SAS Institute, Cary, NC, USA).	patients older than 8 years of age administered with at least one dose of PFA and for whom plasma drug concentration data were available.<br>The somatic efficacy endpoints were analyzed separately in the patients with and without prior ERT with idulsulfase.<br>Regarding HS and DS concentrations in the CSF and neurocognitive testing, the patients with and without prior ERT were analyzed together. In the neurocognitive testing result analyses, age equivalent (AE) scores and developmental quotient (DQ) scores were calculated for the cognitive domain of the BSID III and the nonverbal index of the KABC II. For the VABS II, patients were classified in terms of improvement, stabilization, or deterioration according to the absolute change in

			AE scores for each subdomain at 52 weeks.<br>All statistical analyses were performed with the SAS version 9.4 statistical soft-ware package (SAS Institute, Cary, NC, USA).

Table S2. IRB approvals for the Phase I/II study (JR-141-101 study) in 9 investigational sites in Japan

Investigational site	IRB	Identification No.	Approval date
Kurume University Hospital	Local IRB established by the site	216830	21/ Mar/2017
Saitama Prefectural Children's Medical Center	Local IRB established by the site	29-1	26/April/2017
Osaka City University Hospital	Local IRB established by the site	101930	22/Feb/2017
Gifu University Hospital	Local IRB established by the site	29007	8/May/2017
National Center for Child Health and Development	Local IRB established by the site	A29001	20/April/2017
Jikei University Hospital	Local IRB established by the	28-31（3003）	28/Feb /2017

	site		
Osaka University Hospital	Local IRB established by the site	179006	25/April/2017
Tottori University Hospital	Local IRB established by the site	29-002	22/Mar/ 2017

Table S3. IRB approvals for the Phase II/III study (JR-141-301 study) in 19 investigational sites in Japan

Investigational site	IRB	Identification No.	Approval date
National Center for Child Health and Development	Central IRB (Pediatric Clinical Trials Network)	NW2018104	21/Aug/2018
Osaka University Hospital	Local IRB established by the site	180020-B	24/July/2018
Kurume University Hospital	Local IRB established by the site	218810	17/July/2018
Saitama Prefectural Children's Medical Center	Local IRB established by the site	30-4	10/Oct/2018
Tottori University Hospital	Local IRB established by the site	30-002	25/July/2018
Gifu University Hospital	Local IRB established by the site	30018	6/Aug/2018
Osaka City University Hospital	Local IRB established by the site	102014	25/July/2018
Okayama Medical Center	Central IRB (Pediatric Clinical Trials Network)	NW2018104	21/Aug/2018
Shizuoka Children's Hospital	Central IRB (Pediatric Clinical Trials Network)	NW2018104	21/Aug/2018
Kurashiki Central Hospital	Local IRB established by the site	663	20/Aug/2018
Ryukyu University Hospital	Local IRB established by the site	Heisei 30 No.14	18/Sep/2018
Fujieda Municipal General Hospital	Local IRB established by the site	Not available*	6/Sep/2018
Osaka City General Hospital	Central IRB (Pediatric Clinical Trials Network)	NW2018104	21/Aug/2018

Fukui University Hospital	Local IRB established by the site	2018002	20/Aug/2018
Jichi Medical School Hospital	Central IRB (Pediatric Clinical Trials Network)	NW2018104	21/Aug/2018
Kanagawa Children's Medical Center	Central IRB (Pediatric Clinical Trials Network)	NW2018104	21/Aug/2018
Hokkaido Medical Center	Local IRB established by the site	1809-A-03	5/Sep/2018
Kumamoto University Hospital	Local IRB established by the site	2018002	23/Oct/2018
Fukuoka Children's Hospital	Central IRB (Pediatric Clinical Trials Network)	NW2018104	16/ Oct/ 2018

* Due to the limited number of clinical trials at the site, no ID number was conferred

Table S4. IRB approvals for the Phase II study in two sites in Brazil

Investigational site	IRB	Identification No.	Approval date
Hospital de Clínicas de Porto Alegre	Local IRB established by the site	79886117.6.1001.5327	08/Dec/2017
Instituto de Genética e Erros Inatos do Metabolismo	Local IRB established by the site	79886117.6.2001.5505	07/June/2018
